# Current and future of immunotherapy for thyroid cancer based on bibliometrics and clinical trials

**DOI:** 10.1007/s12672-024-00904-6

**Published:** 2024-02-25

**Authors:** Ke Wang, Ying Zhang, Yang Xing, Hong Wang, Minghua He, Rui Guo

**Affiliations:** 1 Department of Clinical Laboratory, The First Hospital of Jilin University, Jilin University, 1 Xinmin Str, Changchun, 130021 Jilin China; 2 Cancer Center, The First Hospital of Jilin University, Chang Chun, China; 3 College of Computer Science and Technology, Jilin University, Chang Chun, China

**Keywords:** Immunotherapy, Thyroid cancer, Clinical Trials, Scientometric analysis, Immune checkpoint inhibitor, PD-1/PD-L1, Anaplastic thyroid carcinoma (ATC), Medullary thyroid carcinoma (MTC)

## Abstract

**Background:**

Thyroid cancer is a leading endocrine malignancy, with anaplastic and medullary subtypes posing treatment challenges. Existing therapies have limited efficacy, highlighting a need for innovative approaches.

**Methods:**

We analyzed 658 articles and 87 eligible clinical trials using bibliometric tools and database searches, including annual publication and citation trends, were executed using Web of Science, CiteSpace, and VOS Viewer.

**Results:**

Post-2018, there is a surge in thyroid cancer immunotherapy research, primarily from China and the University of Pisa. Of the 87 trials, 32 were Phase I and 55 were Phase II, mostly exploring combination therapies involving immune checkpoint inhibitors.

**Conclusion:**

The study's dual approach verifies the swift advancement of thyroid cancer immunotherapy from diverse perspectives. Immune checkpoint inhibitors have become the preferred regimen for advanced MTC and ATC in late therapeutic lines. However, since ICB plays a pivotal role in ATC, current clinical trial data show that ATC patients account for more and the curative effect is more accurate. Anticipated future developments are inclined toward combination regimens integrating immunotherapy with chemotherapy or targeted therapies. Emerging approaches, such as bispecific antibodies, cytokine-based therapies, and adoptive cell therapies like CAR-T and TCR-T, are exhibiting considerable potential. Upcoming research is expected to concentrate on refining the tumor immune milieu and discovering novel biomarkers germane to immunotherapeutic interventions.

**Supplementary Information:**

The online version contains supplementary material available at 10.1007/s12672-024-00904-6.

## Introduction

Thyroid cancer, a predominant malignancy of the endocrine system, accounts for approximately 1% of all neoplasms. According to the World Health Organization's 2022 classification of thyroid tumors, thyroid cancer is categorized into eight distinct groups: developmental abnormalities, follicular cell-derived neoplasms, thyroid C-cell-derived carcinoma, mixed medullary and follicular cell-derived carcinomas, salivary gland-type carcinomas of the thyroid, thyroid tumors of uncertain histogenesis, thymic tumors within the thyroid and embryonal thyroid neoplasms [1]. Furthermore, within the group of follicular cell-derived neoplasms, there are seven specific subtypes, each with its unique characteristics. These subtypes include: follicular thyroid carcinoma (FTC), invasive encapsulated follicular variant papillary thyroid carcinoma (IEFVPTC), papillary thyroid carcinoma (PTC), oncocytic carcinoma of the thyroid (OCA), poorly differentiated thyroid carcinoma (PDTC), differentiated high-grade thyroid carcinoma (DHGTC), and anaplastic follicular cell-derived thyroid carcinoma (ATC) [1]. The disease notably targets a younger demographic, specifically individuals aged 16–33, and exhibits a higher incidence among females [2]. Key risk factors include a family history of thyroid cancer, Hashimoto's thyroiditis, prolonged exposure to ionizing radiation, excessive iodine intake, and obesity [3–5]. Global incidence rates have shown an uptick, with approximately 568,000 new cases reported in 2020 [6].

Anaplastic thyroid carcinoma stands out as the most aggressive and lethal subtype, characterized by a median survival duration of approximately five months and a one-year overall survival rate of 20% [7, 8]. Traditional therapeutic regimens encompass chemotherapy and radiotherapy, either as monotherapies or in conjunction with surgical intervention. Advances in our molecular understanding have led to the identification of BRAF V600E mutations in about 40% of cases, offering novel therapeutic opportunities through the combined application of BRAF inhibitors like dabrafenib and MEK inhibitors such as trametinib, which have demonstrated significant response rates in BRAF-mutated ATC [9].

Medullary thyroid carcinoma, an exceedingly rare neuroendocrine tumor originating from parafollicular cells, primarily relies on surgical intervention for disease management. Nonetheless, a subset of patients who are either unresectable or experience disease recurrence have limited options, often resorting to targeted or immunotherapeutic modalities [10, 11]. Multi-target tyrosine kinase inhibitors and selective anti-RET tyrosine kinase inhibitors have emerged as efficacious treatments, significantly improving clinical outcomes for these patients [12]. Multiple drugs inhibiting signaling or oncogenic kinases, including those targeting Platelet-Derived Growth Factor Receptor and Vascular Endothelial Growth Factor Receptor, have gained regulatory approval [13].

Despite these advances, the search for additional effective treatments for high-grade thyroid cancers remains an imperative. Immunotherapy has surfaced as a promising strategy, particularly for anaplastic thyroid carcinoma, with diverse approaches ranging from tumor-associated macrophage targeting, cancer vaccines, adoptive immunotherapy, monoclonal antibodies, to immune checkpoint blockade [14]. Notably, the FDA granted approval for the anti-PD-1 antibody pembrolizumab for thyroid cancer treatment in 2020, and clinical trials have substantiated the therapeutic efficacy of the anti-PD-1 antibody spartalizumab in locally advanced or metastatic ATC [11].

Employing bibliometric analysis, a methodology integrating mathematical and statistical approaches for comprehensive literature scrutiny, our study utilizes VOSViewer and CiteSpace software to examine publications on thyroid cancer immunotherapy from 1999 to 2022. We further incorporate an analysis of 86 clinical trials as the cornerstone of our investigation. Our findings affirm that immunotherapy for thyroid cancer is experiencing an acceleration in both the quantity and quality of research, with evolving focal points in the field. As such, a multi-dimensional examination of the current research landscape is warranted.

## Materials and methods

### Scientometric analysis

A database was built to search related literature for bibliometric studies via the Science Citation Index Expanded in Web of Science Core Collection (WoSCC). Medical Subject Headings (MeSH) database was utilized to obtain retrieval terms for the search strategy, TS = ("thyroid cancer" OR "papillary thyroid carcinoma" OR " follicular thyroid cancer " OR "medullary thyroid cancer" OR "anaplastic thyroid cancer" OR "papillary thyroid microcarcinoma")AND TS = ((Immunotherapy) OR (PD-1) OR (PD-L1) OR (CTLA-4) OR (TIGIT) OR (LAG3) OR (TIM-3) OR (A2AR) OR (OX40) OR (ICOS) OR (4-1BB) OR (Cadonilimab) OR (AK-104) OR (Camrelizumab) OR (Nivolumab) OR (Pembrolizumab) OR (Sintilimab) OR (Atezolizumab) OR (Ipilimumab) OR (Durvalumab) OR (Avelumab) OR (Zalifrelimab) OR (Dostarlimab) OR (Balstilimab) OR (gen1042) OR (m7842) OR (SHR-1701) OR (Lifileucel) OR (Antibody Drug Conjugate) OR (Tisotumab vetodik) OR (TNF) OR (IFN) OR (Interleukin) OR (Adoptive Cell Therapy) OR (TIL) OR (TCR-T) OR (CAR-T) OR (CIK) OR (LAK) OR (DC) OR (Therapeutic Vaccine) OR (Therapeutic Vaccination)). The search was limited to English language articles and included articles and reviews published from 1999 to 2023. Finally, this search strategy retrieved 658 records up to January 19, 2023.

VOSViewer 1.6.18 and CiteSpace (6.1.R6) were used to visualize and analyze the data as bibliometric tools and convert the original data into visualization. Then we downloaded 658 results from WoSCC in the Plain Text File form and no duplicate record was found after processing, so there were still 658 records remained. Further, the composition of the database is as follows: there were 654 publications (99.39%) with abstracts, 658 publications (100.00%) with DOI number, 657 publications (99.85%) with subject categories, and 657 publications (99.85%) with cited references. The time range for the analysis was from January 1999 to December 2022, and top 50 levels from each time slice were selected for analysis.

In this study, we analyzed references, and keywords (timeline view analysis and burst analysis) through CiteSpace (6.1.R6). Meanwhile, we used VOS viewer to analyze countries and institutions, keywords and authors of this field. In addition, we chose Google Chart Tools (v1.0) to make a world map, which shows the countries and regions that have made contributions to this topic.

### Clinical trails

In this study, we chose two fundamental data source of clinical trials: ClinicalTrials.gov (https://www.clinicaltrials.gov) and WHO ICTRP (https://trialsearch.who.int). Our search strategy in these databases consisted of the following criteria: 1) Condition or Disease/Condition = ("thyroid cancer" OR "thyroid neoplasms" OR "papillary thyroid carcinoma "OR"follicular thyroid cancer "OR" medullary thyroid cancer" OR "anaplastic thyroid cancer" OR "papillary thyroid microcarcinoma") Other terms/Innervation = (Immunotherapy OR PD-1 OR PD-L1 OR CTLA-4 OR TIGIT OR LAG3 OR TIM-3 OR A2AR OR OX40 OR ICOS OR 4-1BB OR Cadonilimab OR AK104 OR Camrelizumab OR Nivolumab OR Pembrolizumab OR Sintilimab OR Atezolizumab OR Ipilimumab OR Durvalumab OR Zalifrelimab OR Dostarlimab OR Balstilimab OR GEN1046 OR M7824 OR SHR-1701 OR Adoptive Cell Therapy OR TIL OR TCR-T OR CAR-T OR CIK OR LAK OR DC OR Lifileucel OR Antibody Drug Conjugate OR Tisotumab Vetodin OR TNF OR IFN OR Interleukin OR Therapeutic Vaccine OR Therapeutic Vaccination). In total, 97 clinical trials were identified in ClinicalTrials.gov and 48 clinical trials were found in ICTRP. After removing duplicates by Trial IDs, a total of 86 clinical trials were included in our analysis.

## Results

### Scientometric analysis

#### Distribution of publications and citations by year

**Fig. 1:**
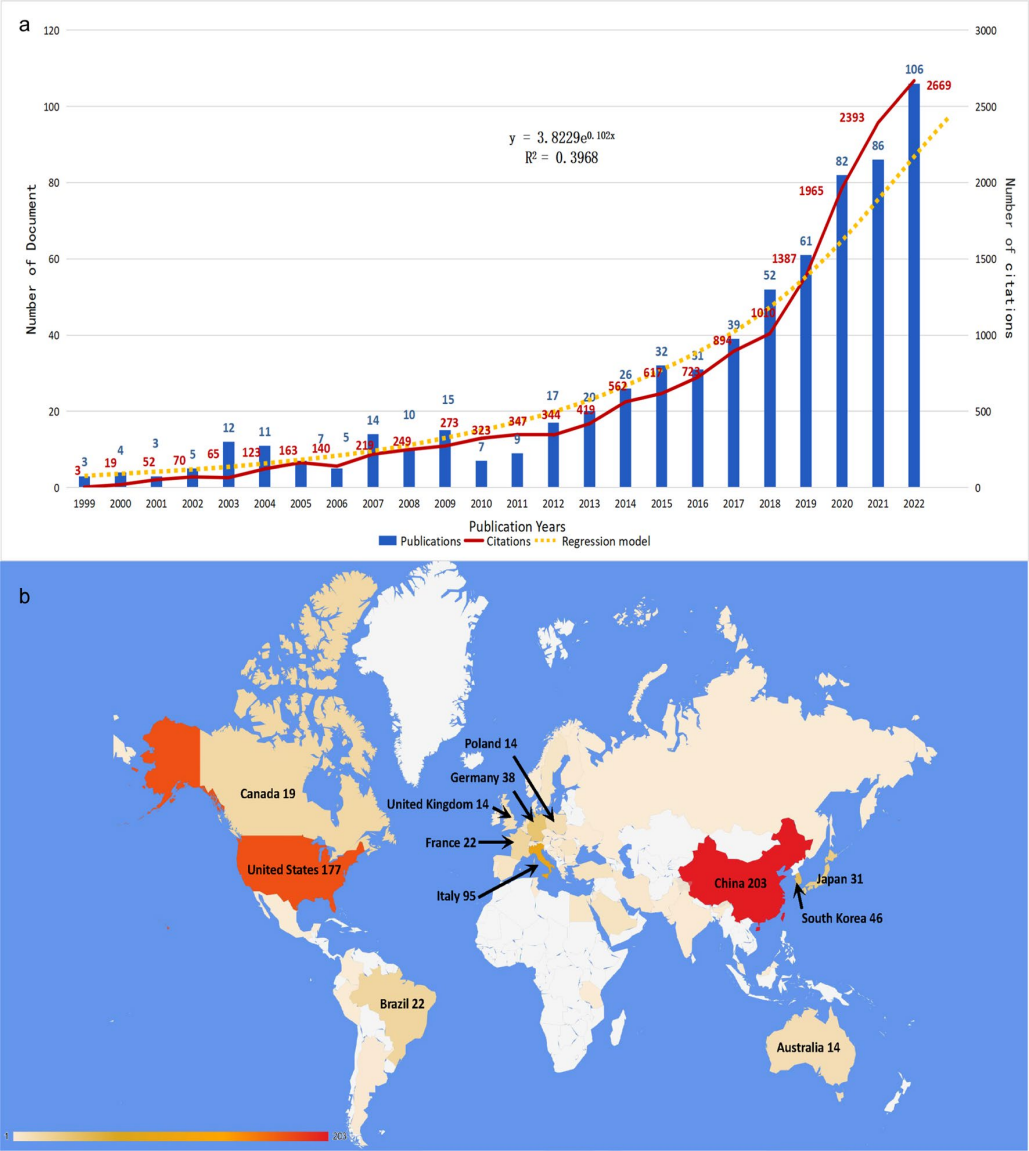
**a** The number of publications and the number of citations by year. The regression model is y = 3.8229e^0.102x^ (R^2^ = 0.3968). **b** The geographical visualization of publications for immunotherapy in thyroid cancer

Based on our comprehensive search in the Web of Science Core Collection, we identified 658 pertinent articles, which collectively garnered 15,029 citations, averaging 22.84 citations per article. As delineated in Fig. 1a, scholarly output in this domain can be bifurcated into two temporal phases: one spanning from 1999 to 2018 and the other from 2019 onward. While the initial phase exhibited a moderate incline in publication frequency, the latter phase saw a pronounced surge in both the number of publications and citations, reflecting heightened scholarly interest. A regression model, y = 3.8229e^0.102x^ (R^2^ = 0.3968), was employed to further elucidate the temporal dynamics of publications.

#### Leading countries

**Fig. 2:**
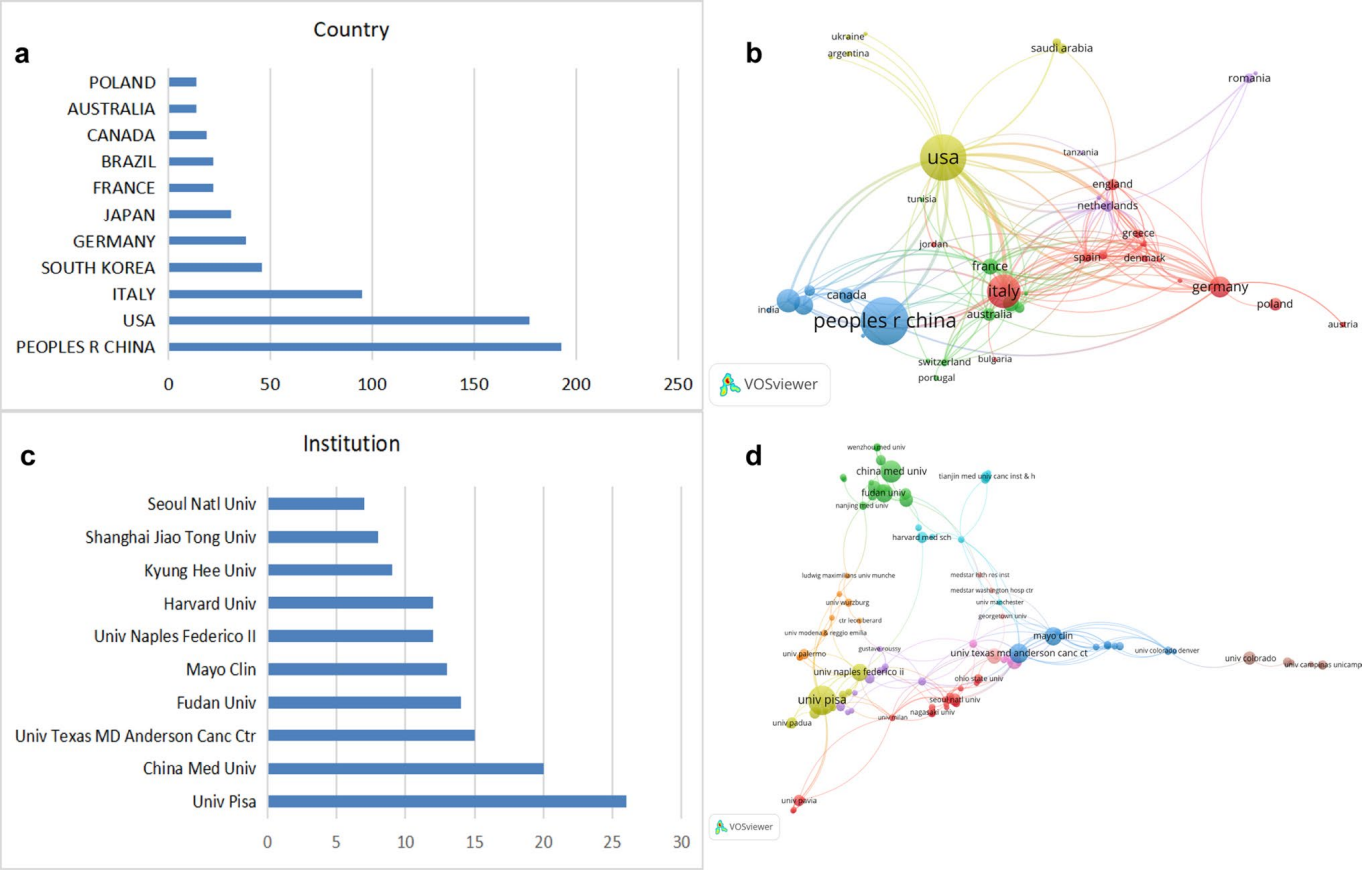
**a** The top 11 countries with the largest number of publications.** b** The map of international cooperation relationship. The size of a node represents the publication number of a country. **c** The top 10 institutions with the largest number of publications. **d** The co-occurrence map of institutions which had related publications in the field of immunotherapy in thyroid cancer

A total of 53 countries and regions have made scholarly contributions to the field of immunotherapy in thyroid cancer, as indicated in Fig. 1b. Notably, 31 of these entities published fewer than five articles. Figure 2a identifies leading contributors, with the People's Republic of China (n = 193), the United States (n = 177), and Italy (n = 95) achieving noteworthy scholarly output. Additionally, these nations have engaged in extensive collaborative research efforts (Fig. 2b).

##### China

China has disseminated 193 articles in the domain of immunotherapy for thyroid cancer. In 2019, out of 233,846 new cases and 45,575 deaths globally, China accounted for 16.71% and 15.88%, respectively [15]. The elevated prevalence of thyroid cancer in specific regions within China is potentially attributable to mild iodine deficiency [16]. Notably, regions such as Guangdong, Zhejiang, and Jiangsu have witnessed high incidence rates particularly among women, with variances of up to 45-fold between the least and most affected areas. Eminent institutions like China Medical University, Fudan University, and Shanghai Jiao Tong University have emerged as leading research centers.

##### USA

In the United States, the incidence of thyroid cancer has escalated more rapidly than any other type of cancer since the 1990s, although a decline has been observed since 2014 [17]. Overdiagnosis is posited as a primary cause for this trend. Among the top-tier institutions contributing to this field are the University of Texas MD Anderson Cancer Center, the Mayo Clinic, and Harvard University.

##### Korea

With a substantial population base, nearly half of thyroid cancer cases are recorded in South and East Asia. Epidemiological evidence suggests that the incidence rate in South Korea has accelerated markedly since the turn of the century. In terms of age-specific incidence rate (ASIR) and age-standardized DALY rate, South Korea ranks highest among the studied regions [18]. Leading academic institutions such as Kyung Hee University and Seoul National University have been prolific publishers in this area. The elevated incidence rates in regions like Kwangju and Jeollanam are correlated with excessive salt intake, primarily derived from seawater evaporation.

#### Leading institutions

Of the 422 institutions contributing to research in immunotherapy for thyroid cancer, 17.04% of the publications emanate from the top 10 institutions, as depicted in Fig. 2c. The co-authorship networks among these institutions are visualized through VOS Viewer (Fig. 2d). These leading institutions comprise three from China, three from the United States, two from Italy, and two from Korea (Table 1). The University of Pisa stands out as the most prolific institution in the study of immunotherapy for thyroid cancer.

**Table 1:** The top 10 institutions

Rank	Institution	Centrality	Publication	cumPubPerc (%)	Country
1	Univ Pisa	0.12	26	3.26	ITALY
2	China Med Univ	0.00	20	5.76	CHINA
3	Univ Texas MD Anderson Canc Ctr	0.10	15	7.64	USA
4	Fudan Univ	0.00	14	9.40	CHINA
5	Mayo Clin	0.01	13	11.03	USA
6	Univ Naples Federico II	0.11	12	12.53	ITALY
7	Harvard Univ	0.06	12	14.04	USA
8	Kyung Hee Univ	0.00	9	15.16	KOREA
9	Shanghai Jiao Tong Univ	0.00	8	16.17	CHINA
10	Seoul Natl Univ	0.02	7	17.04	KOREA

#### Authors

We employed VOS Viewer as our bibliometric tool for a comprehensive analysis of publications and authorship, focusing on citation and co-citation frequencies. Table 2 enumerates the top eight highly productive and highly co-cited authors in the field.

**Table 2:** The top 8 highly productive and highly co-cited authors

Rank	Author	Count	Co-cited Author	Count
1	Antonelli, alessandro	20	Antonelli, alessandro	324
2	Fallahi, poupak	20	Cabanillas, maria e	156
3	Ferrari, silvia martina	18	Cunha, lucas leite	151
4	Ward, laura sterian	9	Schlumberger, m	124
5	French, jena d	8	Subbiah, v	116
6	Haugen, bryan r	8	French, jena d	105
7	Rotondi, mario	8	Brose, ms	102
8	Wang, yu	8	Xing, mz	93

##### High-profile authors

**Fig. 3:**
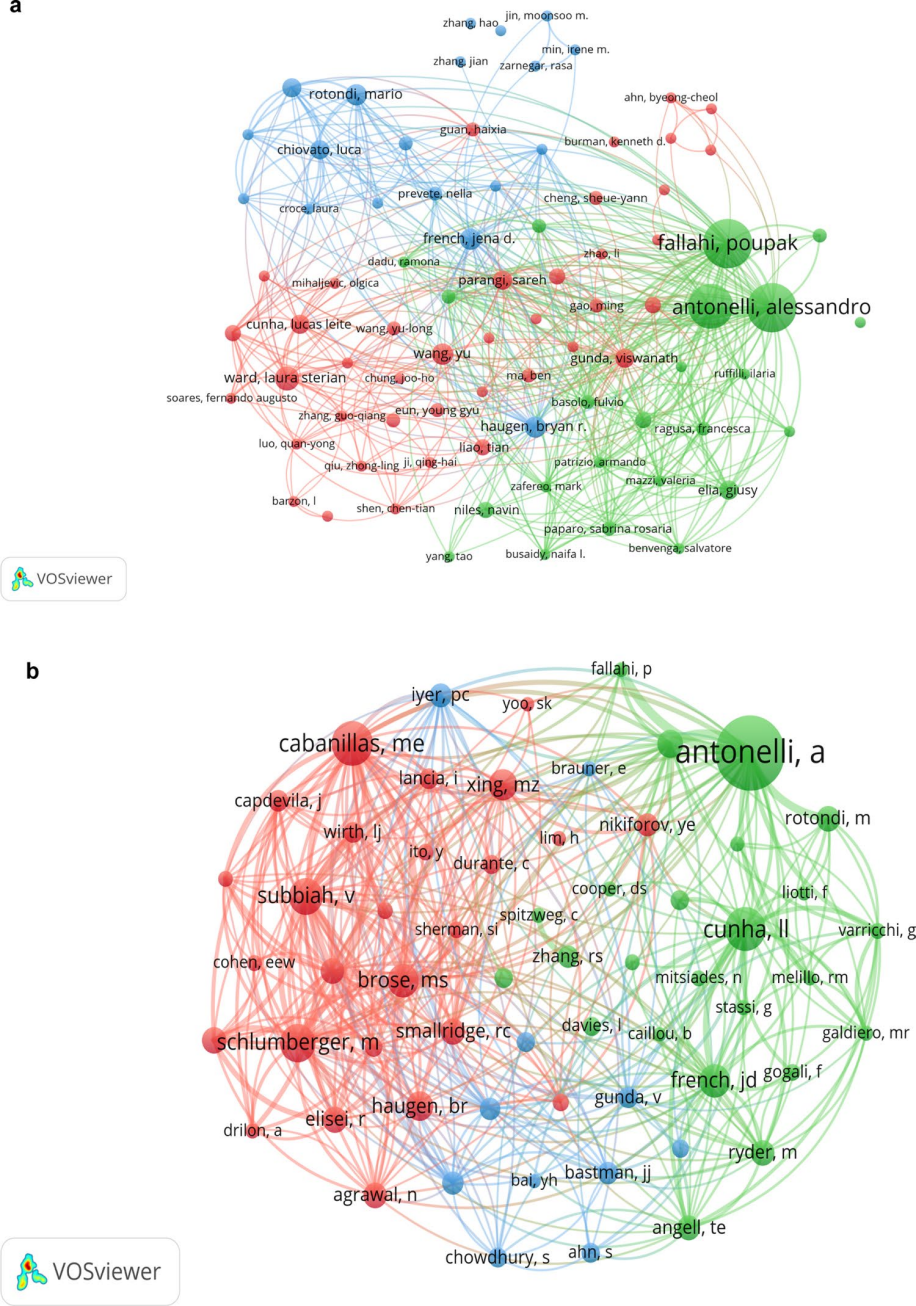
**a** Highly published authors. **b** The network map of researching authors who were co-cited over 30 times

Fourteen authors have published seven or more articles pertaining to immunotherapy in thyroid cancer. Preeminent among these are Fallahi and Antonelli, each with 20 publications, followed by Ferrari (n = 18) and Ward (n = 9). These individuals also represent the most-cited authors in the domain, with Fallahi and Antonelli receiving the highest citation counts (n = 1536), succeeded by Ferrari (n = 1490) (Fig. 3a).

##### Co-citation analysis of authors

In order to show how often these related authors were cited together, a co-citation network, comprising 61 authors who were co-cited at least 30 times, was constructed using VOS Viewer (Fig. 3b). Authors sharing the same color in the network were co-cited with greater frequency. It can also be learned from the figure that there were 21 authors who were co-cited more than or equal to 60 times, and among all the highly co-cited authors, Antonelli, alessandro (n = 324), Cabanillas, maria e. (n = 156) and Cunha, lucas leite (n = 151) were the top 3 authors.

#### Journals

**Fig. 4:**
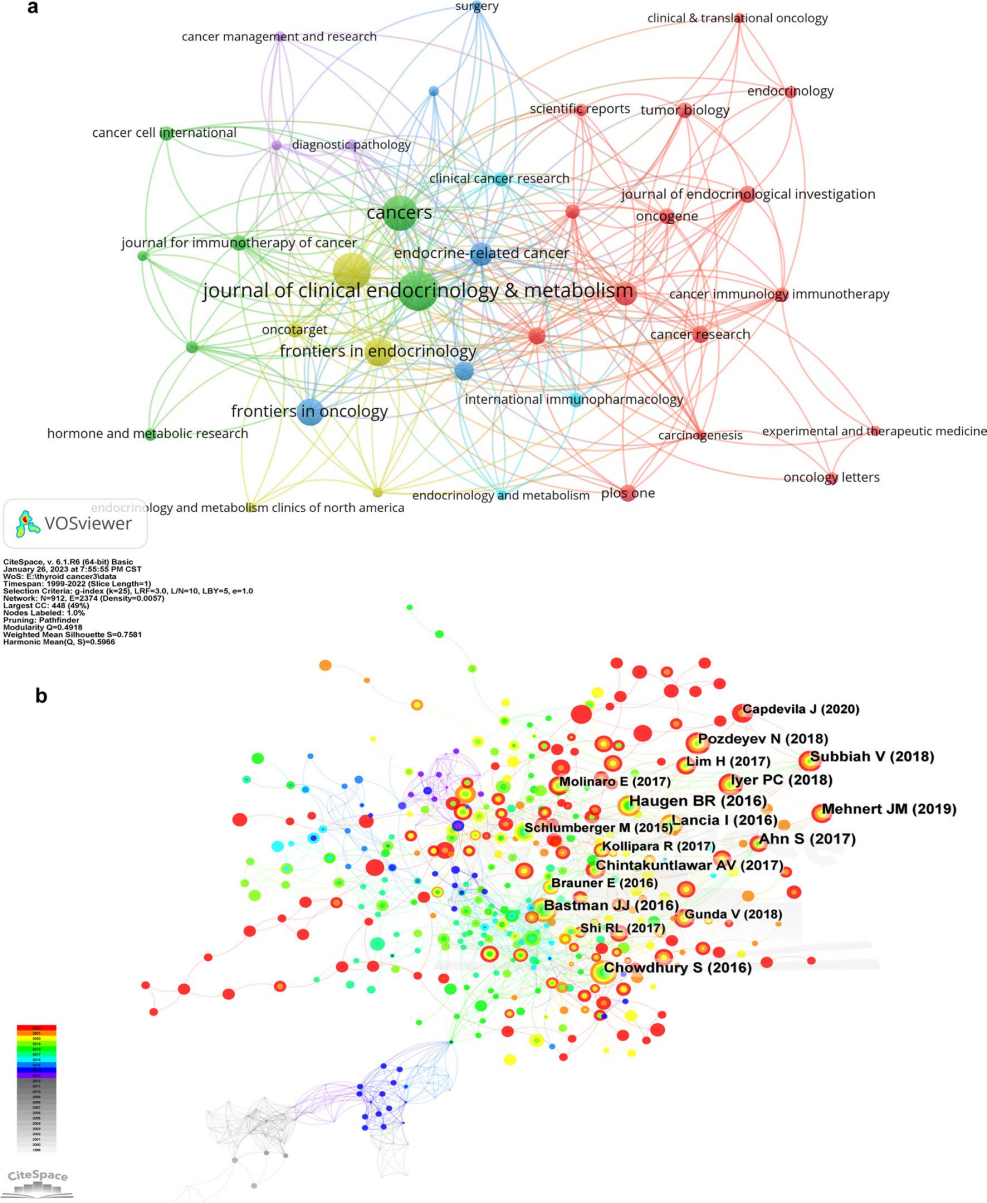
**a** The co-citation analysis of the journals. **b** The co-citation analysis of references

Our analysis identified 297 journals of repute that have published articles in this sphere. The "Journal of Clinical Endocrinology & Metabolism" ranked foremost with 26 publications, followed by "Thyroid" (n = 24) and "Cancers" (n = 22). Inter-journal collaborations were prevalent, as depicted in Fig. 4a. The "Journal of Clinical Endocrinology & Metabolism" also led in citation counts (n = 914), with "Autoimmunity Reviews" (n = 883) and "Thyroid" (n = 669) occupying subsequent ranks, indicating their substantial impact in the field.

#### References

##### Co-citation analysis of the references

To provide an overview of the historical background and research frontiers in the field, we employed Citespace for co-citation analysis (Fig. 4b). The majority of highly co-cited references were published within the last five years, and we made a table of the top ten most co-cited articles (Table 3). Among the ten references, there were eight clinical trials, one review and one comparative Study. Moreover, five clinical trials were associated with ICBs such as PD-1 and PD-L1, one trial was about dabrafenib (BRAF inhibitor) and trametinib (MEK inhibitor), and two trials focused on genetic characterization. Notably, the highest co-cited reference in our analysis was a comprehensive screening for PD-L1 expression in thyroid cancer, indicating the progress that immunotherapy has made in treating thyroid cancer.

**Table 3:** The top 10 co-cited articles

Year	Title	Journal	Type	First author	IF	Central idea	Co-citation
2017	Comprehensive screening for PD-L1 expression in thyroid cancer	ENDOCR- RELAT CANCER	Clinical Trial	Ahn S [19]	4.7704	Evaluated the frequency of PD-L1 expression using a rabbit monoclonal antibody	49
2016	2015 American Thyroid Association Management Guidelines for Adult Patients with Thyroid Nodules and Differentiated Thyroid Cancer	THYROID	Review	Haugen BR [20]	7.7860	Inform clinical decision-making in the management of thyroid nodules and differentiated thyroid cancer	47
2019	Safety and antitumor activity of the anti–PD-1 antibody pembrolizumab in patients with advanced, PD-L1–positive papillary or follicular thyroid cancer	BMC CANCER	Clinical Trial	Mehnert JM [21]	2.9330	Evaluate safety of the PD-1 antibody pembrolizumab in advanced solid tumors	46
2016	Tumor-Infiltrating T Cells and the PD-1 Checkpoint Pathway in Advanced Differentiated and Anaplastic Thyroid Cancer	J CLIN ENDOCR METAB	Clinical Trial	Bastman JJ [22]	5.6050	Whether PD-1 checkpoint inhibitors have a therapeutic effect on patients with invasive thyroid cancer	46
2018	Dabrafenib and Trametinib Treatment in Patients With Locally Advanced or Metastatic BRAF V600-Mutant Anaplastic Thyroid Cancer	J CLIN ONCOL	Clinical Trial	Subbiah V [23]	28.2450	A phase II trial of BRAF inhibitor and MEK inhibitor combination therapy in BRAF V600E-mutated anaplastic thyroid cancer	43
2018	Salvage pembrolizumab added to kinase inhibitor therapy for the treatment of anaplastic thyroid carcinoma	J IMMUNOTHER CANCER	Clinical Trial	Iyer PC [24]	8.6760	The curative effect of adding pembrolizumab to kinase inhibitors at progression in ATC	43
2016	Genomic and transcriptomic hallmarks of poorly differentiated and anaplastic thyroid cancers	J CLIN INVEST	Clinical Trial	Lancia I [25]	12.2820	Genetic characterization of poorly differentiated and undifferentiated thyroid carcinoma	42
2016	Programmed death-ligand 1 overexpression is a prognostic marker for aggressive papillary thyroid cancer and its variants	ONCOTARGET	Compara- tive Study	Chowdhury S [26]	5.1680	Analyzed PD-L1 expression in PTC and its variants and determined its prognostic potential to predict clinical outcome in these patients	40
2017	Expression of PD-1 and PD-L1 in Anaplastic Thyroid Cancer Patients Treated With Multimodal Therapy: Results From a Retrospective Study	J CLIN ENDOCR METAB	Clinical Trial	Chintakuntlawar AV [27]	5.6050	Detect potential associations of PD-1/PD-L1 axis variables with outcome data in ATC	36
2018	Genetic Analysis of 779 Advanced Differentiated and Anaplastic Thyroid Cancers	CLIN CANCER RES	Clinical Trial	Pozdeyev N [28]	8.9110	Identify potential diagnostic, prognostic, and therapeutic significance of the genetic landscape for advanced differentiated and anaplastic thyroid cancer	34

##### Co-citation timeline view of references

**Fig. 5:**
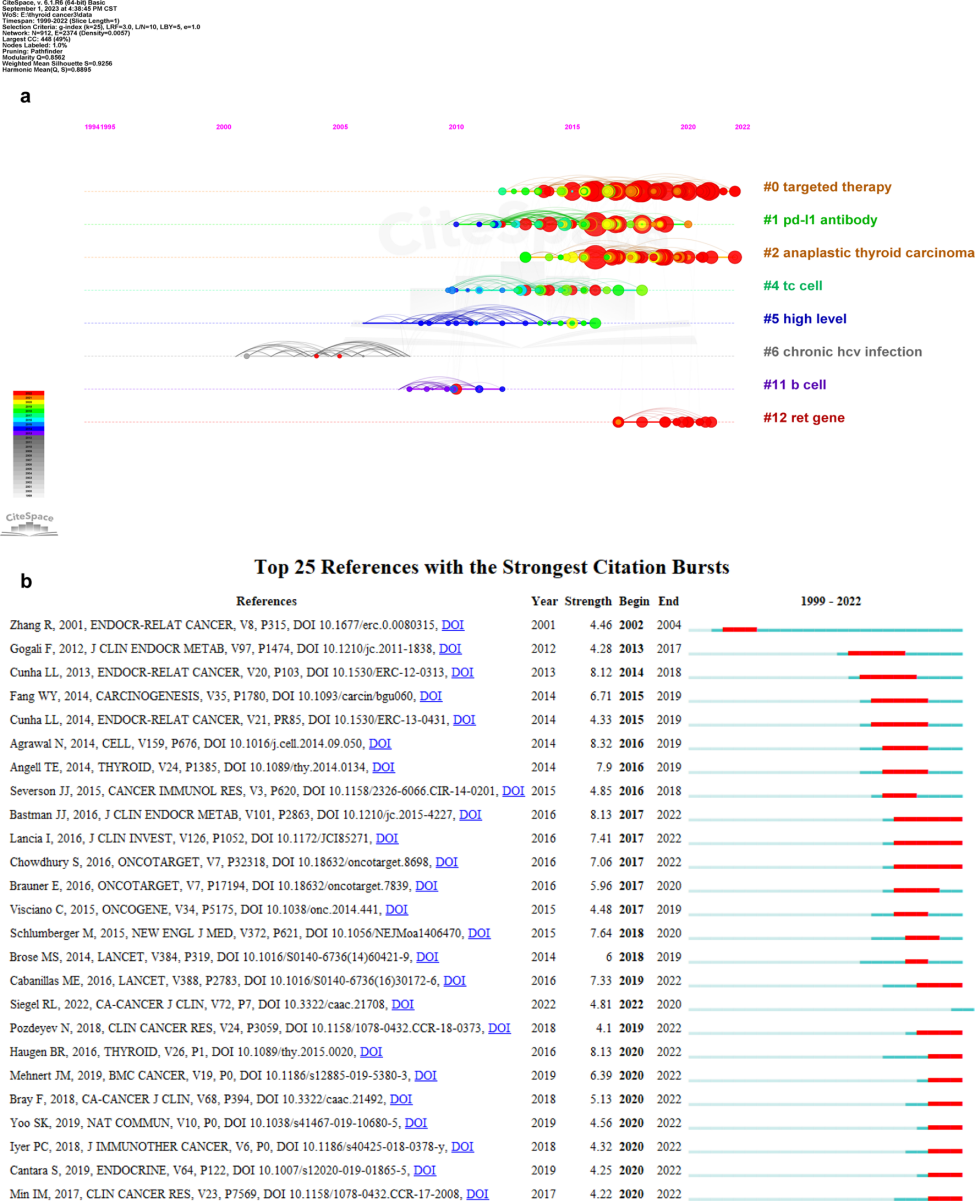
**a** The time axis view of references. **b** The high reference outbreak analysis

Different nodes in the reference co-citation timeline view represent different references, and the larger the node, the greater the influence of the reference. On the time axis, the years of different references are displayed from left to right, and the nodes closer to the right represent the newer references. The clustering in the Fig. 5a also further shows some hot topics in the field of thyroid cancer immunotherapy, such as #0 target therapy and #1 PD-L1 antibody.

##### Burst analysis of references

Bibliometric burst analysis shows us the relationship between the citation of some references and time (Fig. 5b). The position of the red line segment on the right represents the duration of the outbreak of the reference. This shows us the direction and evolution of thyroid cancer immunotherapy over time, which can help us speculate on the future development prospects of this field.

#### Co-occurrence analysis of keywords

**Fig. 6:**
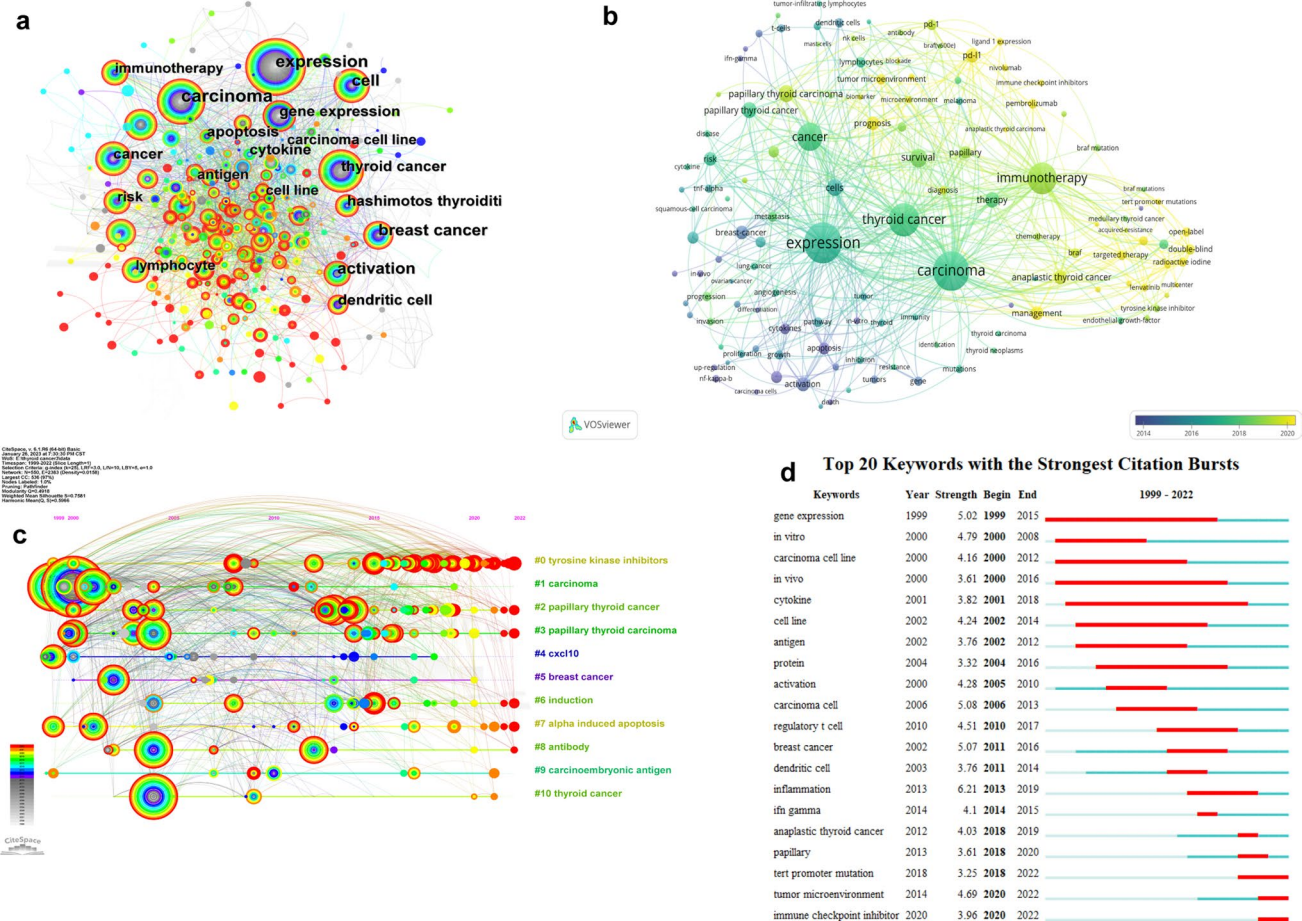
**a** The co-occurrence map of the keywords with high frequency.** b** The overlay visualization of keywords. The average year of keyword by assigning different colors to the nodes. **c** The timeline view of keywords from Citespace. The top 11 clusters were selected in our analysis. **d** The top 20 keywords with the strongest citation bursts from Citespace

As the core vocabulary, keywords serve as the cornerstone in encapsulating the primary focus of a research paper, and are also the high-frequency vocabulary in the article. To discover the main content of immunotherapy for thyroid cancer and the relationship between keywords, we made the keyword co-occurrence map (Fig. 6a, b) through Citespace and VOSviewer.

A total of 116 keywords emerged with frequencies of 11 or greater. We selected these 116 keywords and divided them into 4 clusters. Table 4 lists the top ten keywords with the highest frequency. Expression (n = 183), carcinoma (n = 173), thyroid cancer (n = 139), immunotherapy (n = 128) are the four keywords which have the highest frequency. It is obvious from Fig. 6b that the largest circles reflected the main content of our study: the immunotherapy for thyroid cancer. In addition, Fig. 6b also shows the process of the development of the keywords we selected over time. The icon in the lower right corner shows that the closer the color is to yellow, the closer it is to 2022, and the closer it is to purple, the farther it is from now in time.

**Table 4:** The top 10 keywords with the highest frequency

Rank	Keyword	Count
1	Expression	183
2	Carcinoma	174
3	Thyroid cancer	162
4	Cancer	90
5	Papillary thyroid carcinoma	61
6	Cell	57
7	Survival	51
8	Papillary thyroid cancer	47
9	Breast cancer	44
10	Anaplastic thyroid cancer	42

#### Timeline view of keywords

Using Citespace, we created a timeline view which captures the development and evolution of a topic (Fig. 6c). After clustering all the keywords, it generated several clusters with different sizes. Further, each cluster comprises multiple closely related words, with the smaller clusters having more keywords. We selected 11 clusters in our study:, #1 carcinoma, #2 papillary thyroid cancer,#3 papillary thyroid carcinoma, #4 cxcl 10,#5breast cancer, #6 induction, #7 alpha induced apoptosis, #8 antibody, #9 carcinoembryonic antigine and #10 thyroid cancer.

The figure can be divided roughly into two periods: early and current stages. The clusters such as #0 tyrosine kinase inhibitors was the hottest topic in immunotherapy for thyroid cancer at present, indicating that TKIs (tyrosine kinase inhibitors) are the most principal targeted therapy for thyroid cancer.

#### Burst analysis of keywords

CiteSpace's burst analysis of keywords allows us to detect changes in reference amounts over a specific period, thus enabling us to understand the process of development and decline of related keywords. In order to show the strength of the burst and the change of research focus, we use CiteSpace to generate keyword burst diagrams (Fig. 6d). The top20 burst keywords were selected and the initial research directions were as follows: the strongest one is gene expression (5.02), followed by in vitro (4.79) and carcinoma cell line (4.16). Further, gene expression appeared early (1999) and immune checkpoint inhibitor appeared at latest (2020). The strength of the keywords which represented immunotherapy such as immune checkpoint inhibitor and tert promoter mutation were not very high. However, a large number of new keywords have emerged recently, which shows that the research topics of immunotherapy in thyroid cancer are developing and updating rapidly.

##### Cytokine

Cytokine is the fifth in Fig. 6d and ranked 43 in the co-occurrence analysis. Further, there are also some keywords that related to cytokine: IFN gamma, cxcl10, interleukin 6, interleukin 8 and so on. Cytokine first occurred in 2001 and last for 18 years which is the keyword that lasts the longest time. Cytokine therapy has a glorious past for it is the earliest immunotherapy drug approved by the FDA for tumors. Till now, it still has value to be explored but has basically matured.

##### Tyrosine kinase inhibitors

From Fig. 6c, #0 tyrosine kinase inhibitors is the first cluster, indicating that it contains the largest number of keywords. Ranking 19 in the co-occurrence analysis means that TKI is a commonly used immunotherapy for thyroid cancer. So far, at least 58 receptor tyrosine kinases (RTKs) and 32 non-receptor tyrosine kinases (NRTKs) have been identified [29]. For advanced thyroid cancers, anti-angiogenic tyrosine kinase inhibitor drugs are the major targets [30]. Tyrosine kinase inhibitors are continuously being developed as potential drugs in the future for the treatment of thyroid cancer.

##### Immune checkpoint inhibitor

As showed in Fig. 6d, immune checkpoint inhibitor is the 20th in the burst analysis of keywords and ranked 46 in the co-occurrence analysis and many related keywords also appear recently. Immune checkpoint inhibitors can eliminate tumors by preventing the expression of immune checkpoint molecules on tumors, such as PD-1 or PD-L1, so that they can be recognized by lymphocytes. In economically developed countries, ICBs therapy has been used in nearly half of patients with metastatic cancer. Till December 2021, there are 8 approved drugs available for 17 different malignancies [31]. Ipilimumab is the first ICB to enter clinical use and was approved by the FDA in 2011. In the clinical trial part of our study, 7 clinical trials related to ipilimumab were included, mostly in combination with nivolumab. Further, there are 16 clinical trials used pembrolizumab in our study which was approved by FDA in 2020, as a result the keywords immune checkpoint first appeared in 2020 and continued to nowadays. The application of immune checkpoint blockers has opened up a brand-new path for thyroid cancer treatment and whether single or several immune checkpoint inhibitors or combined with other treatment is still the most worth exploring immunotherapy method.

## Clinical trials

### Development and current status of related clinical trials

**Fig. 7:**
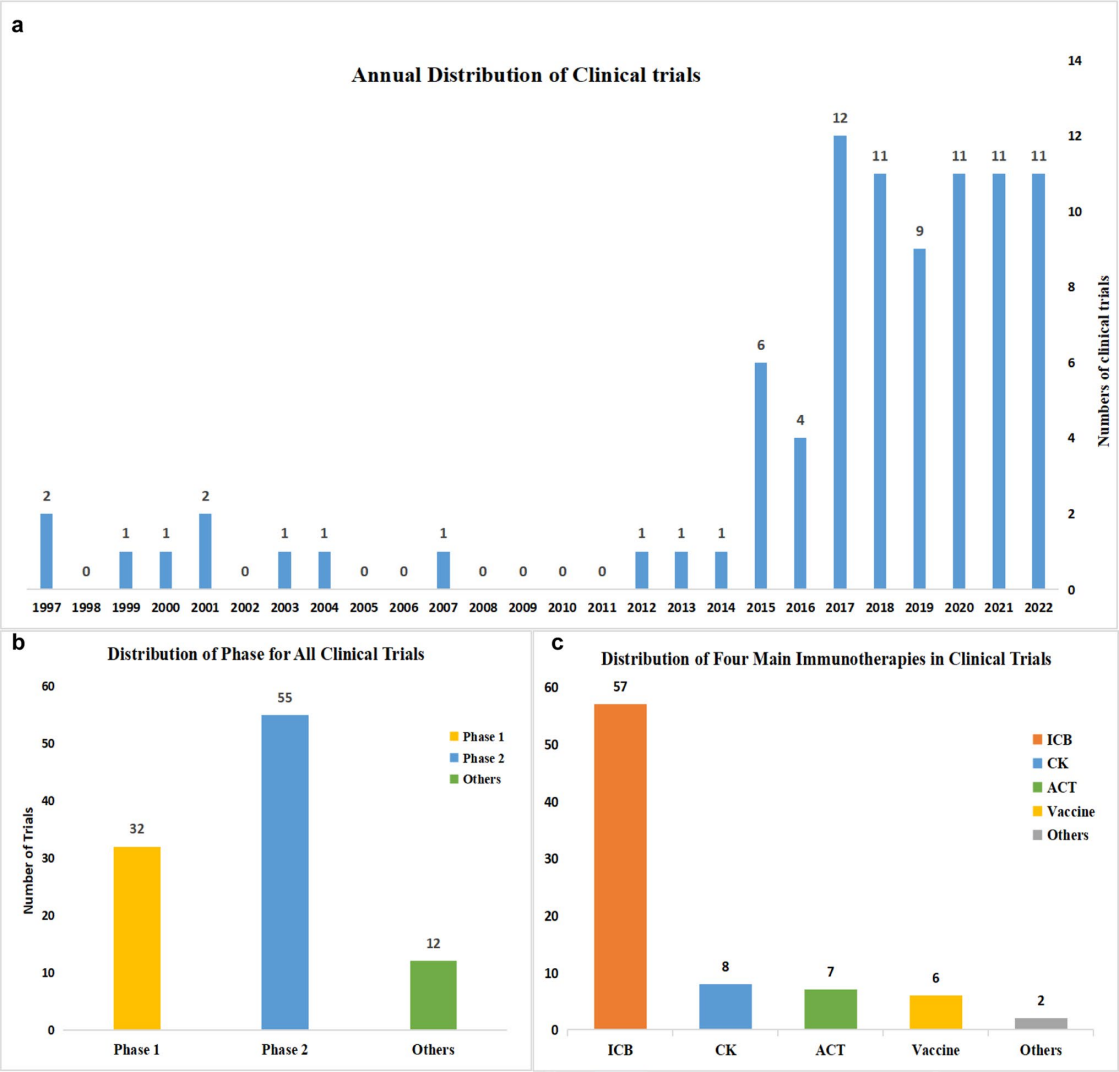
**a** The annual distribution of clinical trials from 1997 to 2022. The start date was regarded as the time of clinical trials. **b** The distribution of phases for all clinical trials.** c** The distribution of the main types of immunotherapies in all clinical trials

We conducted a comprehensive analysis of 87 clinical trials and recorded the NCT number, title, status, interventions, study type, start date, phases, treatment mode and detail of treatment (Additional file 1). It was witnessed that Immunotherapy in thyroid cancer started on February 1, 1997: a clinical trial using CEA RNA-pulsed DC cancer vaccine treating patients with metastatic cancer (NCT00004604). We analyzed annual distribution of clinical trials (Fig. 7a). It was found that during 1997 to 2014, there were only a few clinical trials and the number of clinical trials had risen sharply after 2015,indicating that Immunotherapy in thyroid cancer have drawn more and more attentions. In 2017, the number of clinical trials reached maximum (n = 12).

There were 32 clinical trials of Phase I and 55 clinical trials of Phase II (Fig. 7b). Most of the clinical trials were Phase II, which shows the therapeutic effect and safety of drugs on patients with target indications. It shows that immunotherapy in thyroid cancer has been widely explored recently.

#### Immunotherapy strategies of related clinical trials

**Fig. 8:**
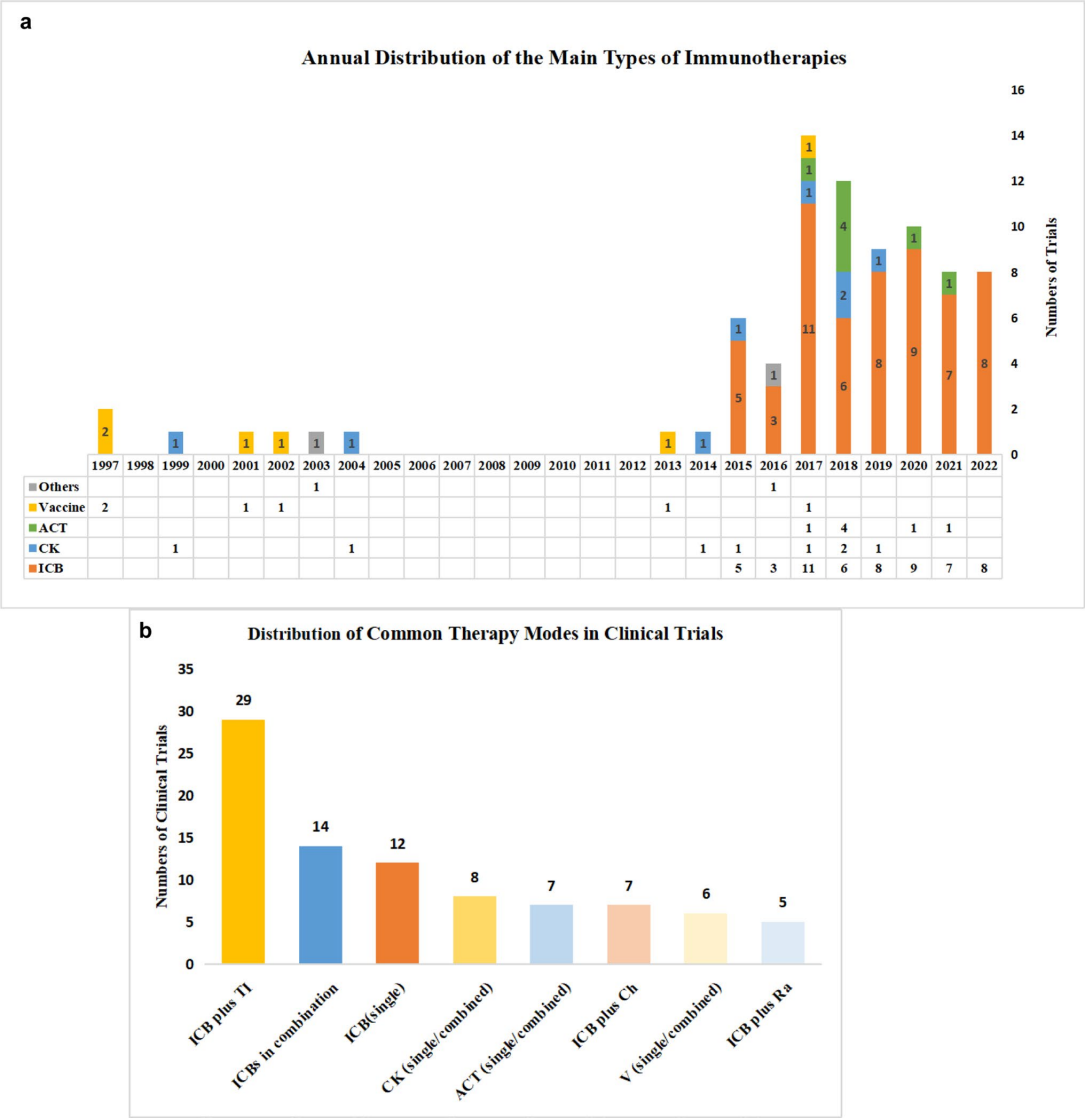
**a** The annual distribution of the main types of immunotherapies including ICB, CK, ACT, and Vaccines from 1997 to 2022. **b** The distribution of some common combined therapies. ICB plus TI referred to ICB combined with targeted therapy. ICBs in combination referred to several ICBs that were used simultaneously in a clinical trial. CK (single/combined) referred to CK that was used as a single agent or combined with other therapies. ACT (single/combined) referred to ACT that was used as a single agent or combined with other therapies. V (single/combined) referred to vaccines that were used as a single agent or combined with other therapies. ICB plus Ch referred to ICB combined with chemotherapy. ICB plus Ra referred to ICB combined with radiotherapy

We examined the various immunotherapy strategies being utilized in clinical trials for thyroid cancer and classified them into four main categories: immune checkpoint blockage (ICB), vaccine, adoptive cell therapy (ACT) and cytokine (CK). Through the distribution (Fig. 7c) and annual distribution (Fig. 8a) of immunotherapies, we found that ICB has been the main immunotherapy in thyroid cancer for it has the highest frequency (n = 57). In the early years, vaccine was the most concerned. However, as time goes on, CK (n = 8) and ACT (n = 7) gained more and more attention. Especially, all of the ACT therapies occurred after 2017, indicating a growing interest in this advanced immunotherapy approach.

Furthermore, we identified eight common treatment options for immunotherapy in thyroid cancer, of which involved single or combination immunotherapies (Fig. 8b). The combination of ICB and targeted therapy was the commonest (n = 29), followed by ICBs in combination (n = 14) and single ICB (n = 12). CK (n = 8) and ACT (n = 7) are also getting more and more popular. Some well-known and traditional treatment, such as ICB combined with chemotherapy (n = 7) or radiotherapy (n = 5) were also the common therapies.

#### Clinical trials of some important immunotherapeutic drugs

According to our analysis of clinical trial data, there were a total of 17 clinical trials related to the anti-PD-1 immune checkpoint blocker (ICB) pembrolizumab, 15 of which were in phase II and 2 in phase I. Notably, 13 of these clinical trials were initiated after 2017, indicating an increasing interest in pembrolizumab. This ICB is often combined with Lenvatinib (a tyrosine kinase inhibitor) or chemotherapy in clinical trials. Another anti-PD-1 ICB, nivolumab, was the subject of 16 clinical trials, including 14 in phase II and 4 in phase I. Furthermore, there are 7 clinical trials concerned with the combination of nivolumab and ipilimumab (anti-CTLA-4 ICB). For anti-PD-L1 ICB atezolizumab, there are 6 clinical trials in phase II (three phase I/II). Only 1 clinical trial used vudalimab as bispecific antibodies (PD-1, CTLA-4).

As a branch of tumor immunotherapy and an important direction for future medical development, adoptive cell therapy (ACT) includes several approaches, such as CAR-T, TCR, TIL and so on. After 2017, there were 7 clinical trials related to ACT: 4 single CAR-T therapies, 1 CAR-T combined with chemotherapy and 2 TIL combined with anti-PD-1 ICB.

## Discussion

### Epidemiological status of thyroid cancer

The cornerstone of treatment for most patients diagnosed with well-differentiated thyroid cancer (WDTC), particularly those of low-risk categorization, remains surgical resection. Nonetheless, individuals exhibiting high-risk attributes often require a more nuanced therapeutic continuum, incorporating elements such as thyrotropin (TSH) suppression and radioiodine therapy (RAI) [32]. The advent of immunotherapy in recent years heralds a transformative epoch in the management of thyroid malignancies. Immunotherapeutic strategies fundamentally aim to modulate endogenous immune responses or employ targeted pharmaceutical agents with well-defined objectives for antineoplastic effects. Notably, immunotherapy is predominantly employed for subtypes resistant to conventional treatments.

Among the spectrum of thyroid malignancies, medullary thyroid cancer (MTC) constitutes a minority, representing approximately 3–5% of cases [32]. The therapeutic axiom for MTC involves the identification and subsequent targeting of actionable mutational events. Additionally, high tumor mutational burden serves as a selection criterion for immunotherapeutic intervention, while cases lacking such criteria generally default to standard chemotherapy. Thus, the penetration of immunotherapy within the realm of MTC remains restricted. Anaplastic thyroid carcinoma (ATC), although rare, is the most aggressive variant of thyroid cancer, characterized by a median survival duration ranging between 5 and 12 months and a one-year survival rate of 20–40% [33]. These grim prognostic metrics render immunotherapy a pivotal frontier for therapeutic exploration in ATC.

Our investigation, employing both scientometric analysis and review of clinical trials, substantiates the accelerated development of immunotherapeutic modalities in thyroid cancer. A detailed analysis of the implications of these findings follows.

### Targeted therapy for MTC and ATC

As our comprehension of the genetic and molecular underpinnings of thyroid malignancies deepens, targeted therapies for specific genetic mutations have evolved concomitantly. For instance, BRAF-V600E serves as a diagnostic cornerstone across multiple pathological subtypes of thyroid cancer and correlates with the therapeutic effectiveness of BRAF inhibitors such as vemurafenib and dabrafenib [34, 35]. Medullary thyroid carcinoma (MTC) frequently harbors mutations in the proto-oncogene RET [36]. Agents targeting this gene, such as selpercatinib and pralsetinib, have shown promise for MTC patients harboring these specific mutations [37]. The NTRK gene family includes NTRK1, NTRK2 and NTRK3. NTRK gene fusion is a carcinogenic driver for various tumor types [38]. NTRK gene fusion is very common in some rare tumors, but also occurs in solid tumors, but the probability is very small, which includes thyroid cancer [39]. In response to such genetic abnormalities, new targeted drugs have been designed to treat related malignant tumors. Anaplastic lymphoma kinase (ALK) is found in a subtype of anaplastic large cell lymphoma. The types and number of ALK-positive tumors are increasing year by year, including anaplastic thyroid carcinoma, non-small cell carcinoma and so on [40]. Targeting ALK through RNA interference, monoclonal antibodies, small molecule inhibitors, has benefited patients in personalized cancer treatment [40, 41].

### Chemotherapy for MTC and ATC

Chemotherapy remains a critical modality in the treatment of both MTC and ATC. Dacarbazine, a frequently utilized chemotherapeutic agent, has demonstrated efficacy in MTC and shows enhanced effectiveness when combined with adjunctive therapies such as radiotherapy or other chemotherapeutic agents like cyclophosphamide, vincristine, and 5-fluorouracil [42, 43]. Docetaxel is a chemotherapeutic drug that interferes with cell classification by interfering with microtubule networks. Docetaxel has been shown to play an active role in the treatment of ATC, and thyroid tumors are significantly inhibited after combined treatment with radiotherapy [44].

### Microenvironment of thyroid cancer immunotherapy

The occurrence, growth and metastasis of tumors are closely related to the internal and external environment of tumor cells, including surrounding blood vessels, various cells, signal molecules and extracellular matrix. Under homeostatic conditions, immune surveillance mechanisms serve to maintain cellular equilibrium, including the elimination of nascent tumor cells through intricate immunological pathways. When cancer cells appear in the body, NK cells first kill them, and dendritic cells will extract new tumor antigens to T cells, and then produce corresponding antibodies through the cooperation of T cells and B cells [45]. Conversely, tumor cells often employ a myriad of strategies to evade immunological detection, including the recruitment of immunosuppressive cells and the modulation of their own immunogenicity [46]. Myeloid-derived suppressor cells (MDSC) are immature bone marrow cells, which are significantly elevated in patients with thyroid cancer and can exhibit strong immunosuppressive effects [47]. In the process of thyroid cancer cells escaping from immune surveillance, the presence of MDSC and M2 macrophages can help cancer cells avoid the killing of the immune system [48].

### Immunotherapy markers for thyroid cancer

As tumor treatment enters the stage of precise immunotherapy, it is necessary to find biomarkers to guide the use of immunotherapy. Programmed cell death 1 (PD-L1) is an important immunosuppressive molecule that promotes tumor growth by inhibiting the activation of T cells [49]. It can inhibit the proliferation and activation of T cells by activating PD-1 and weakening the key dephosphorylation process in T cell receptor pathway, and can further change its metabolism and function, and even lead to the death of T cells [50]. Notably, the BRAF^V600E mutation has been shown to induce PD-L1 expression, making it a viable immunotherapeutic target in thyroid cancer [51]. Tumor mutation burden (TMB) serves as a quantitative measure of genetic mutations within tumors. The higher the value of TMB is, the better the effect of ICB treatment will be [52]. Whole exome sequencing showed that thyroid cancer had the third lowest TMB (between 0.1 and 1 mut / Mb) across 27 tumors [53]. In recent years, the research on TMB has gradually matured, and it has also become a potential marker and was approved by the FDA in 2020. Especially when combined with PD-L1, it has been shown to be a marker available in certain cancer types.

### Immunotherapy

#### Immune checkpoint blockades

Immune checkpoint blockades have inaugurated a paradigm shift in cancer immunotherapy. These checkpoints serve as pivotal immunoregulatory elements, safeguarding against autoimmunity while maintaining immune homeostasis [37]. Currently, Programmed Death Receptor-1 (PD-1), Programmed Death Ligand-1 (PD-L1), and Cytotoxic T Lymphocyte-Associated Antigen 4 (CTLA-4) are the most extensively investigated checkpoints [30]. Beyond these canonical checkpoints, our study also encompasses LAG-3 and TIGIT (NCT05347212 and NCT05286801). Our analysis includes 57 clinical trials concerning immune checkpoint blockades, many of which employ combination therapies for thyroid cancer.

##### Single immune checkpoint blockade

Our investigation identified 12 clinical trials (13.95%) utilizing a singular immune checkpoint blockade and all of them were phase II clinical trials. These trials span from the earliest, initiated in 2015 (NCT02404441), to the most recent, initiated in February 2022 (NCT05119296). Of these, 11 trials opted for anti-PD-1 antibody as an immune checkpoint blockade, while one chose PD-L1 as therapeutic target (EUCTR2015-000269–30-DE). Three of them have already concluded (NCT02404441,NCT02688608,NCT03072160) and with spartalizumab and pembrolizumab as the selected agents. NCT02404441 included patients with advanced malignant tumors, including melanoma, non-small cell lung cancer, triple negative breast cancer and anaplastic thyroid cancer. NCT02688608 and NCT03072160 chose pembrolizumab in the treatment of metastatic or locally advanced ATC and recurrent or metastatic MTC. Preliminary safety evaluations have been completed for these inhibitors.

##### Multiple immune checkpoint blockades

Cytotoxic T-lymphocyte antigen 4 (CTLA-4) and programmed cell death-1 (PD-1) are the most commonly used immune checkpoint inhibitors [54]. In our cohort, 14 clinical trials (16.28%) employed a combination of two checkpoint inhibitors, either alone or in conjunction with other therapeutic modalities: four in synergy with targeted therapy, two with chemotherapy and one with adoptive cell therapy. These trials were primarily phase II studies, with three classified as phase I. Various combinations of checkpoints were explored, but the majority (9 trials) chose PD-1 in tandem with CTLA-4. PD-L1 and TIGIT (NCT05286801), PD-L1 and CTLA-4 (NCT03122496),PD-1 and PD-L1 (NCT04400474),double PD-1 (NCT05659186) were selected for the remaining four clinical trials.

##### Immune checkpoint blockades combined with tyrosine kinase inhibitor

In our study, there are 29 clinical trials using one or two ICB combined with targeted therapies and 15 (17.24%) of them used tyrosine kinase inhibitor (TKI), such as famitinib, lenvatinib, surufatinib, encorafenib and cabozantinib (Table 5). With the emergence of sorafenib which is the first TKI approved by the FDA in November 2013 and is used for the treatment of advanced metastatic DTC which is ineffective with RAI treatment [55], numerous of clinical trials accured. Among them, the combination of ICB and TKI, such as some fixed treatment modes such as Cabozantinib and Atezolizumab(PD-L1), Cabozantinib and Nivolumab(PD-1) and Ipilimumab(CTLA-4), Lenvatinib and Pembrolizumab(PD-1) are still feasible ideas for the treatment of thyroid cancer.

**Table 5:** The clinical trials using ICB combined with tyrosine kinase inhibitor

Rank	NCT Number	Title	Status	Start Date	Drugs	PFS	ORR
1	NCT02973997	Lenvatinib and Pembrolizumab in Differentiated Thyroid Cancers (DTC)	Active, not recruiting	2018/2/7	Lenvatinib + Pembrolizumab(PD-1)	12 months	No result
2	NCT05696548	Nivolumab Plus Lenvatinib Against Anaplastic Thyroid Cancer (NAVIGATION)	Recruiting	2019/7/2	Lenvatinib + Nivolumab(PD-1)	No study results posted Completion:2025–07	
3	ChiCTR1900026894	A Phase II Study to Explore the Safety and Efficacy of Famitinib Combined with Camrelizumab in the Treatment of Advanced Thyroid Cancer	Recruiting	2019/10/31	Famitinib + Camrelizumab (PD-1)	No study results posted Completion:2022–10-31	
4	NCT04521348	Multiple Target Kinase Inhibitor and Anti-Programmed Death-1 Antibody in Patients With Advanced Thyroid Cancer	Recruiting	2019/12/30	mTKI + PD-1	No study results posted Completion:2024–06-30	
5	NCT04524884	Toripalimab Combined With Surufatinib for Locally Advanced Thyroid Cancer: a Phase II Study	Unknown status	2020/10/1	Surufatinib + Toripalimab(PD-1)	No study results posted Completion:2022–09-30	
6	NCT04061980	Encorafenib and Binimetinib With or Without Nivolumab in Treating Patients With Metastatic Radioiodine Refractory BRAF V600 Mutant Thyroid Cancer	Recruiting	2020/10/30	Encorafenib + Nivolumab(PD-1)	No study results posted Completion:2026–10-30	
7	NCT04514484	Testing the Combination of the Anti-cancer Drugs XL184 (Cabozantinib) and Nivolumab in Patients With Advanced Cancer and HIV	Recruiting	2020/11/4	Cabozantinib S-malate + Nivolumab(PD-1)	No study results posted Completion:2025–11-02	
8	NCT04579757	Surufatinib in Combination With Tislelizumab in Subjects With Advanced Solid Tumors	Active, not recruiting	2021/3/5	Surufatinib + Tislelizumab(PD-1)	No study results posted Completion:2024–06-30	
9	NCT04171622	Lenvatinib and Pembrolizumab for the Treatment of Stage IVB Locally Advanced and Unresectable or Stage IVC Metastatic Anaplastic Thyroid Cancer	Recruiting	2021/11/21	Lenvatinib + Pembrolizumab(PD-1)	No study results posted Completion:2025–08-31	
10	NCT03170960	Study of Cabozantinib in Combination With Atezolizumab to Subjects With Locally Advanced or Metastatic Solid Tumors	Active, not recruiting	2017/9/5	Cabozantinib + Atezolizumab(PD-L1)	No study results posted Completion:2024–08	
11	NCT04400474	Trial of Cabozantinib Plus Atezolizumab in Advanced and Progressive Neoplasms of the Endocrine System. The CABATEN Study	Recruiting	2020/10/7	Cabozantinib + Atezolizumab(PD-L1)	No study results posted Completion:2024–03	
12	NCT02496208	Cabozantinib S-malate and Nivolumab With or Without Ipilimumab in Treating Patients With Metastatic Genitourinary Tumors	Active, not recruiting	2015/7/9	Cabozantinib S-malate + Nivolumab(PD-1) + Ipilimumab(CTLA-4)	No study results posted Completion:2024–09-30	
13	NCT03914300	Testing the Combination of Cabozantinib, Nivolumab, and Ipilimumab (CaboNivoIpi) for Advanced Differentiated Thyroid Cancer	Active, not recruiting	2019/7/15	Cabozantinib + Nivolumab(PD-1) + Ipilimumab(CTLA-4)	No study results posted Completion:2024–01-15	
14	NCT03866382	Testing the Effectiveness of Two Immunotherapy Drugs (Nivolumab and Ipilimumab) With One Anti-cancer Targeted Drug (Cabozantinib) for Rare Genitourinary Tumors	Recruiting	2019/4/12	Cabozantinib + Nivolumab(PD-1) + Ipilimumab(CTLA-4)	No study results posted Completion:2024–02-29	
15	EUCTR2017-004570–34-DE	Phase II clinical trial for the combination of Lenvatinib and Pembrolizumab in patients with anaplastic- or poorly-differentiated thyroid carcinomas	Authorised	2018/11/15	Lenvatinib + Pembrolizumab(PD-1)	No study results posted	

Among these trials, Cabozantinib is the most widely used TKI (n = 6), followed by Lenvatinib (n = 4). In the realm of ICB, 13 selected anti-PD-1 drugs were utilized. Regarding the combination of these two therapies, Lenvatinib combined with Pembrolizumab emerges as the most frequently employed combination, and it consistently yields clear experimental results across all clinical trials. Therefore, we speculate that Lenvatinib combined with Pembrolizumab should be considered a more effective combination.

The ATLEP trial, registered under EUCTR2017-004570–34-DE and conducted by the Medical Center—University of Freiburg in Germany, is an integral part of our statistical analysis. As illustrated in Table 5, this trial falls under the category of a phase II clinical trial. Its primary objective is to assess the efficacy of the combined regimen of lenvatinib and pembrolizumab in patients diagnosed with poorly differentiated thyroid carcinoma and anaplastic thyroid cancer. As our research progresses, the synergistic effects of this combination therapy are becoming increasingly evident, leading to a rise in the objective response rate (ORR). This study serves to further underscore the unique advantages of utilizing lenvatinib and pembrolizumab in the treatment of thyroid cancer. Currently, this clinical trial is ongoing, with plans for an extensive evaluation of the safety and efficacy of immune checkpoint blockade (ICB) combined with tyrosine kinase inhibitor (TKI) treatment. We eagerly anticipate favorable experimental outcomes that will lend substantial support to the adoption of this combination therapy.

**Fig. 9:**
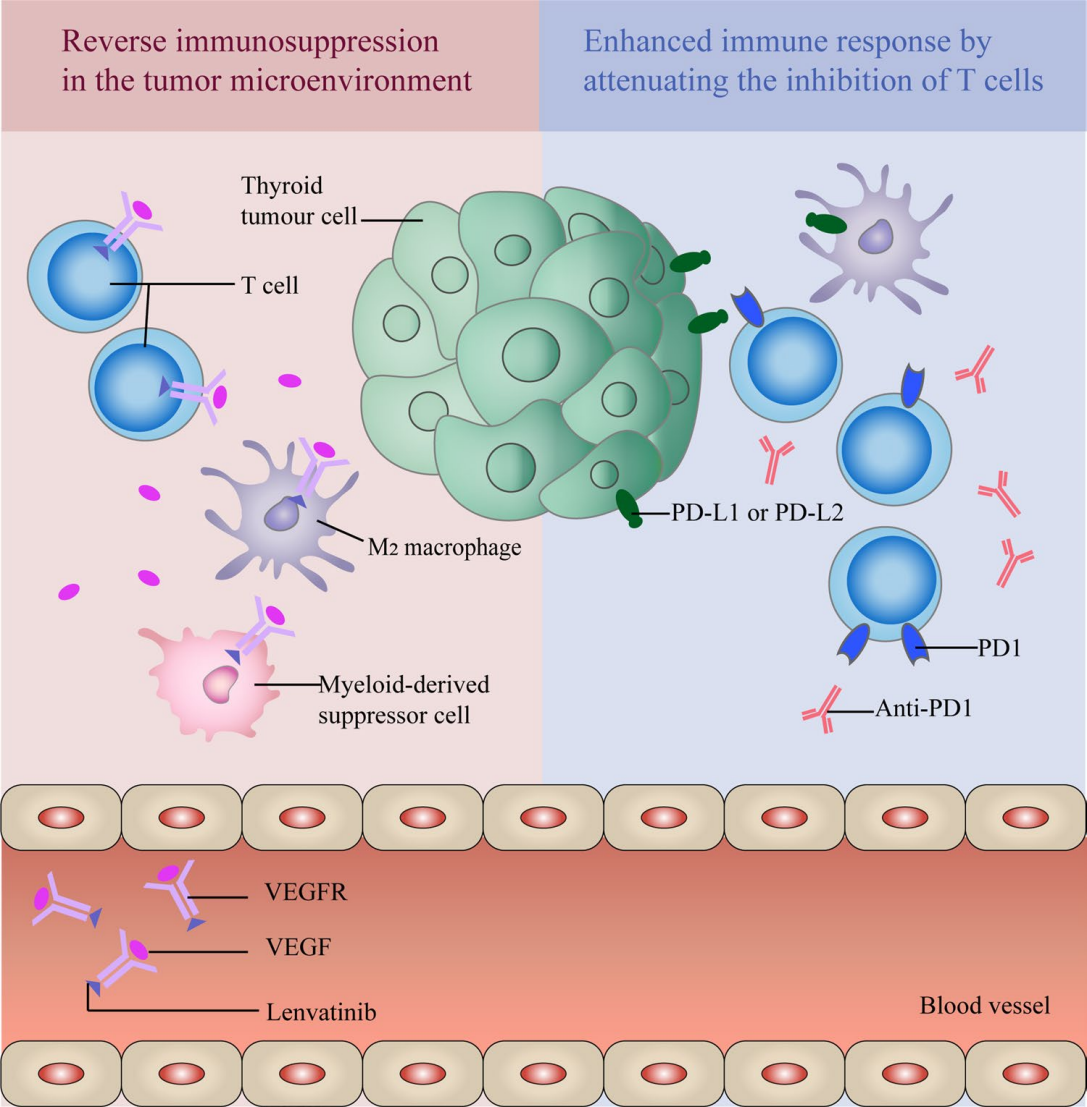
The mechanism of PD-1 immune checkpoint inhibitor combined with lenvatinib (TKI) in the treatment of thyroid cancer

IBC combined with TKI is the most promising treatment, and related clinical trials account for more than half of the number of immune checkpoint combined with targeted therapy. Studies have shown that the expression of PD-L1 in ATC was higher than that in other subtypes [56]. In addition, BRAF V600E mutation may lead to higher PD-L1 mRNA expression. Therefore, PD-L1 expression is proposed as a possible biomarker for immunotherapy. Figure 9 shows the common combination types in thyroid cancer immunotherapy, anti-PD-1 immune checkpoint inhibitors and tyrosine kinase inhibitor (lenvatinib). On the one hand, ICB regulates the immune system and promotes its attack on tumor cells by attenuating the inhibition of T cells. On the other hand, lenvatinib slows down tumor growth by blocking VEGF or VEGFR pathways and reducing blood vessels. In addition, VEGF inhibition can also reverse the phenomenon of immunosuppression in the tumor microenvironment by inhibiting regulatory T cells, M2 macrophages and myeloid-derived suppressor cells (MDSC).

##### Immune checkpoint blockades combined with chemotherapy

Chemotherapy remains an essential pillar of cancer treatment. Together with surgery and radiotherapy, they are called the three major treatments for cancer. In our dataset,, there were 7 clinical trials (8.14%) combined with immune checkpoint blockades and chemotherapy. Because of the wide application of chemotherapy, most clinical trials have combined a variety of treatment methods, so 4 of the 7 clinical trials also combined with targeted therapy, one combined with radiotherapy. NCT03211117 was a phase II clinical trials using pembrolizumab, chemotherapy, and radiotherapy to treat patients with ATC. The clinical trials in our study show that docetaxel and doxorubicin hydrochloride are drugs which are usually used in chemotherapy. They can stop the tumor cell growth through a variety of ways, including cell killing, cell division inhibition, and metastasis prevention.

##### Immune checkpoint blockades combined with radiotherapy

Radiotherapy utilizes high-energy X-rays to shrink tumors and eradicate tumor cells [57]. In the treatment of thyroid cancer, radiotherapy is also a commonly used treatment method, such as iodine 131 treatment for differentiated thyroid cancer. In addition, radiotherapy can be more accurate by targeting molecules that are preferentially expressed on cancer cells. It is usually used when residual cancer cells still exist or cancer cells have spread to other sites after surgical removal of the thyroid gland [58]. Our study identified five clinical trials (5.81%) combining immune checkpoint inhibitors with radiotherapy (NCT03122496, NCT05659186, NCT04675710, NCT03215095, NCT03211117). These combinations, particularly the triad of pembrolizumab, chemotherapy, and radiation therapy, may offer enhanced therapeutic outcomes for patients with anaplastic thyroid cancer.

#### Adoptive cell therapy

Adoptive cell therapy (ACT) entails the isolation, amplification, and functional characterization of immunocompetent cells from patients with cancer, followed by their reinfusion into the patient to target neoplastic cells. Within the scope of our study, ACT is represented in 7 clinical trials: 4 exclusively utilize chimeric antigen receptor T-cell (CAR-T) therapies (4.65%), 1 combines CAR-T with chemotherapy (1.16%), and 2 engage tumor-infiltrating lymphocytes (TILs) alongside anti-PD-1 immune checkpoint blockade (ICB) (2.33%).

##### Tumor infiltrated lymphocyte (TIL)

TIL therapy is an emerging modality of cell-based immunotherapy that employs autologous immune cells harvested from the tumor microenvironment for therapeutic purposes [59]. Ontrasted with other immunotherapeutic strategies such as CAR-T and ICBs, TILs offer advantages in multi-receptor targeting, enhanced tumor tropism, and reduced adverse events. In our investigation, two clinical trials (2.33%) deployed TILs in combination with anti-PD-1 ICBs. Specifically, LN-145 and LN-145-S1 were used in conjunction with ipilimumab and nivolumab in a Phase II trial focusing on anaplastic thyroid cancer (NCT03449108). However, outcomes remain pending, necessitating further research.

##### Chimeric antigen receptor T-cell (CAR-T)

CAR-T therapy is an evolving form of immunotherapy that involves the genetic modification of T-cells to express specific chimeric antigen receptors (CARs), enabling targeted cytotoxicity against tumor cells [60]. Our study identified that ICAM1 (20%), GFRa4 (20%), and TSHR (60%) were the principal targets (NCT04420754, NCT04877613, ChiCTR1800014944, ChiCTR1800014936, ChiCTR1800017612). However, 4 of them are in phase I and only 1 is in phase I/II, indicating that CAR-T in the treatment of thyroid cancer is not mature enough so it still has a long way to go.

##### TCR-T, CAT-NK or NK or CIK

To enhance the efficiency of T-cell-mediated cancer cell eradication, T-cells are engineered in vitro to express tumor-specific T-cell receptors (TCR-T) [61]. TCR-T therapy is still in the process of development due to the high manufacturing cost and complex process, and the difficulty of improving T cell persistence [61, 62]. Compared with CAR-T therapy, CAR-NK has more advantages, such as higher safety and manufacturing feasibility [63]. Natural Killer (NK) cell therapy deviates from T-cell strategies by obviating the necessity for major histocompatibility complex (MHC) presentation, offering increased safety and control and also has higher heat in the current adoptive cell therapy [64]. Cytokine-induced killer (CIK) cells are another variant of T-cells, easily harvested and capable of MHC-unrestricted tumor cell lysis [65]. Therefore, whether they are used alone or in combination with other immunotherapy, they have broad prospects. Unfortunately, the above several adoptive cell therapies have not appeared in our clinical trials, but experiments and studies have shown that they are effective in the treatment of solid tumors. It is believed that there will be more clinical trials of these strategies for the treatment of thyroid cancer in the future.

##### Vaccine

Our study identified a total of 6 clinical trials (6.98%) that incorporate cancer vaccines; four were in Phase I and two in Phase II. Notably, the majority of these trials were initiated prior to 2015, with the exception of one (NCT03127098), which began in 2017 and combines a cancer vaccine with the cytokine IL-15. Vaccines for the treatment of cancer are mainly divided into peptide- and protein-based vaccines, cellular vaccines, genetic vaccines and other types of cancer vaccines [66]. NCT00004604 is a clinical trial of CEA RNA-pulsed DC cancer vaccine in the treatment of metastatic cancer, and it is also the earliest clinical trial in our study. Despite four decades of active research in this realm, the clinical translation of cancer vaccines remains limited. Continuous innovation, possibly in combination with other forms of immunotherapy, holds the promise of yielding more effective treatments [66]. It is believed that through continuous improvement tumor vaccines will achieve greater results.

#### Cytokine

Cytokines, as a class of small molecular proteins with multifarious biological activities, are synthesized and secreted by a variety of immune and non-immune cells. They serve dual roles within the tumor microenvironment, either fostering or inhibiting tumorigenesis [67]. Based on different functions, cytokines can be divided into interleukin (ILs), interferon (IFNs), colony-stimulating factors (CSFs), tumor necrosis factors(TNFs) and chemokines. Accordingly, cytokine-targeted immunotherapies can be divided into two overarching categories: cytokine-directed therapies and anti-cytotoxic factor therapies.

##### Monotherapy with Single Cytokine

Among the clinical trials exploring cytokine applications, 3 trials (3.49%) employed monotherapy using a single cytokine (NCT00098943, NTR7487, EUCTR2017-003028–59-NL). Specifically, Anakinra, a recombinant, non-glycosylated form of human interleukin-1 receptor antagonist, was chosen for treating anaplastic thyroid carcinoma in the latter two studies. NCT00098943 is a phase I clinical trial started in 2004, and CNGRC peptide-TNF alpha conjugate was selected. A number of studies have shown that interleukin is a cytokine that can affect the proliferation of thyroid cancer cells [67]. Particularly, IL-1α and IL-1β, members of the IL-1 family, have undergone extensive studies with respect to malignancies. IL-1α has been implicated in tumor-promoting activities, including tumor dedifferentiation and lymphangiogenesis [68, 69]. Conversely, while IL-1β has been shown to possess anti-tumor cell proliferative effects, its impact on ATC remains undefined [67]. Consequently, both trials in our dataset opted for anti-IL-1 therapies for ATC management.

##### Cytokine combination therapy

In our analysis, 5 clinical trials (5.81%) utilized cytokines in combination with other therapeutic modalities, such as immune checkpoint inhibitors, targeted therapies, chemotherapy, and radiation and vaccine therapies (NCT03127098, NCT04234113, NCT00004074, NCT02516774, NCT02390739). Compounds such as SO-C101, combined with immune checkpoint blockade pembrolizumab, and ALT-803, used in tandem with ETBX-011 vaccine, are associated with IL-15 activation. Furthermore, IL-12, IL-2, and TNF-α were employed in various phase I and II clinical trials.

### The future of immunotherapy in thyroid cancer

While surgical resection remains the preferred treatment for resectable thyroid cancer, it is not without its challenges, such as high recurrence rates and potential damage to nerves in the neck, including the recurrent laryngeal nerve. Additionally, the use of iodine-131 in thyroid cancer treatment also carries associated risks, including gastrointestinal reactions, neck swelling, and parotid gland injury. However, the advent and continuous advancement of immunotherapies, particularly immune checkpoint inhibitors, have begun to alter the therapeutic landscape for various malignancies, including advanced and recurrent thyroid cancers. Immunotherapy, particularly immune checkpoint blockade, is at the center of this progress. Targeted therapies of thyroid cancer have been widely used in clinical practice. As discussed earlier, the combination of ICB and targeted drugs, particularly tyrosine kinase inhibitors, is a crucial direction for future development in thyroid cancer immunotherapy.

#### Strengths of the study

Our research presents a thorough analysis of the current state of immunotherapy in thyroid cancer, emphasizing the characteristics and emerging trends in this rapidly evolving field. We offer a robust reference for both clinicians and researchers, featuring meticulous, objective, and comprehensive insights. The statistical analysis of clinical trials constitutes a significant aspect of our research, and a comprehensive review of the strategies for immunotherapy of thyroid cancer based on statistical results. Finally, based on the above two points, we speculate the future development trajectory and research focus in this field.

#### Limitation of the Study

In our study, although we have conducted a comprehensive search for thyroid cancer immunotherapy as far as possible, the literature we collected may not be comprehensive due to the limitation of search strategy and the limitation of year and literature type. Moreover, the constraints of available visualization tools and statistical methods may not entirely represent the academic consensus. Finally, as far as clinical trials are concerned, it is a pity that the number of relevant clinical trials is small, and although there has been progress in thyroid cancer treatment with immunotherapy, our study shows that most of existing clinical trials are in phase I or II and many are still recruiting participants. Therefore, there is a need for a larger number of randomized, controlled phase III clinical trials to support these findings. Nevertheless, despite these limitations, our study provides invaluable insights that will inform future research in thyroid cancer immunotherapy.

### Supplementary Information

**Additional file 1.** Lists information about 87 clinical trials included in the study, including NCT number, title, status, interventions, study type, start date, phases, treatment mode and detail of treatment.

## Data Availability

The original contributions presented in the study are included in the article/additional material, and further inquiries can be directed to the corresponding author.

## Abbreviations

WoSCC: Web of Science Core Collection
MeSH: Medical Subject Headings
TS: Topic subject
ICTRP: International Clinical Trials Registry Platform
FTC: Follicular thyroid carcinoma
PTC: Papillary thyroid carcinoma
ATC: Anaplastic thyroid carcinoma
MTC: Medullary thyroid carcinoma
ICB: Immune checkpoint blockade
TKI: Tyrosine kinase inhibitor
NCT: National clinical trial
DC: Dendritic cell
CK: Cytokine
ACT: Adoptive cell therapy
CAR-T: Chimeric antigen receptor T cell
TCR: T cell receptor
TIL: Tumor infiltrated lymphocyte
WDTC: Well-differentiated thyroid cancer
TSH: Thyrotropin
RAI: Suppression and radioiodine therapy
ASIR: Age specific incidence rate
DALY: Disability-adjusted life year
FDA: Food and Drug Administration
RTKs: Receptor tyrosine kinases
NRTKs: Non-receptor tyrosine kinases
IL: Interleukin
IFN: Interferon
ALK: Anaplastic lymphoma kinase
MDSC: Myeloid-derived suppressor cells
CSF: Colony-stimulating factor
TNF: Tumor necrosis factors
PD-1: Programmed cell death receptor-1
PD-L1: Programmed cell death ligand-1
CTLA-4: Cytotoxic T lymphocyte-associated molecule-4
LAG-3: Lymphocyte-activation gene 3
TIGIT: T cell immunoglobulin and ITIM domain
